# Development and Quality of Barley Husk Adhesion Correlates With Changes in Caryopsis Cuticle Biosynthesis and Composition

**DOI:** 10.3389/fpls.2019.00672

**Published:** 2019-05-24

**Authors:** Maree Brennan, Pete E. Hedley, Cairistiona F. E. Topp, Jenny Morris, Luke Ramsay, Steve Mitchell, Tom Shepherd, William T. B. Thomas, Stephen P. Hoad

**Affiliations:** ^1^Scotland’s Rural College, Edinburgh, United Kingdom; ^2^James Hutton Institute, Dundee, United Kingdom; ^3^Institute of Molecular Plant Sciences, University of Edinburgh, Edinburgh, United Kingdom

**Keywords:** barley (*Hordeum vulgare*), caryopsis, cementing layer, grain development, grain skinning, husk adhesion, plant cuticle

## Abstract

The caryopses of barley become firmly adhered to the husk during grain development through a cuticular cementing layer on the caryopsis surface. The degree of this attachment varies among cultivars, with poor quality adhesion causing “skinning”, an economically significant grain quality defect for the malting industry. Malting cultivars encompassing a range of husk adhesion qualities were grown under a misting treatment known to induce skinning. Development of the cementing layer was examined by electron microscopy and compositional changes of the cementing layer were investigated with gas-chromatography followed by mass spectroscopy. Changes in gene expression during adhesion development were examined with a custom barley microarray. The abundance of transcripts involved early in cuticular lipid biosynthesis, including those encoding acetyl-CoA carboxylase, and all four members of the fatty acid elongase complex of enzymes, was significantly higher earlier in caryopsis development than later. Genes associated with subsequent cuticular lipid biosynthetic pathways were also expressed higher early in development, including the decarbonylation and reductive pathways, and sterol biosynthesis. Changes in cuticular composition indicate that lowered proportions of alkanes and higher proportions of fatty acids are associated with development of good quality husk adhesion, in addition to higher proportions of sterols.

## Introduction

Barley grains have an outer husk composed of two bract-like structures, the lemma and palea, which in cultivars used for malting adhere to the caryopsis (fruit) during the later stages of grain filling and development (Harlan, 1920; Hoad et al., 2016; Brennan et al., 2017a). Husk adhesion is known to be facilitated by a cuticular cementing layer produced by the pericarp, which is the fruit coat on the caryopsis surface (Harlan, 1920; Gaines et al., 1985). During grain development, the cementing layer feels sticky to the touch, and adhesion occurs when the caryopsis nears maximum volume, coming into contact with the husk organs. The physical and chemical processes that mediate husk adhesion have not yet been fully determined. It is known, however, that the quality of husk adhesion can be compromised, resulting in an economically significant grain quality defect known as “skinning.” Skinning, the loss of some or all of the husk at harvest, has been shown to adversely affect the efficiency of the malting process, and the homogeneity of the malt produced (Meredith, 1959; Okoro et al., 2017). Understanding the process of husk adhesion, and the causes of differential adhesion qualities, is an important step toward grain quality improvement in malting barley.

Recently, we have shown that there are genotypic differences in the quality of husk adhesion among cultivars of malting barley, and that under controlled conditions adhesion can be influenced by multiple environmental factors (Brennan et al., 2017a,b). Within the commercially important cultivar Concerto, growth at different temperatures during husk and caryopsis development resulted in a range of husk adhesion qualities (Brennan et al., 2017a). It had previously been postulated that differing grain dimensions could result in mis-matched mechanical strengths between husk and caryopsis tissues (Rajasekaran et al., 2004; Hoad et al., 2016). The study of Brennan et al. (2017a), however, demonstrated that within one cultivar changes in adhesion quality were correlated with altered abundance of some compounds in the cementing layer, rather than layer thickness or grain dimensions. As only one cultivar was examined in that study, it is not clear whether the differences in husk adhesion quality among genotypes is also correlated with changes in the composition or thickness of the cementing layer, or grain and organ dimensions. One environmental treatment, misting to simulate rainfall during grain filling, has not been fully investigated to determine whether this effect is correlated with grain physiology or changes in the lipid cementing layer on the caryopsis surface.

Barley cultivars that do not have adherent husks also exist, and are known as “naked” or “hulless” barley. This phenotype is distinct from the poor quality adhesion which causes skinning, as naked barleys do not produce a lipid cementing layer on the caryopsis surface, only a cuticle on the pericarp (fruit coat) of the caryopsis (Gaines et al., 1985; Brennan et al., 2017a). The covered or naked phenotype of barley is determined by the presence or absence of a functional NUD protein. Naked barleys have a 17-kb deletion at the *nudum* (*NUD*) locus on chromosome 7H, resulting in loss of function (Taketa et al., 2008). The *NUD* gene is homologous to the *Arabidopsis WIN1/SHN1* transcription factor which is thought to regulate cuticular lipid biosynthesis (Aharoni et al., 2004; Broun et al., 2004; Kannangara et al., 2007). A comparative study of naked and covered barley using RNA-seq has indicated that *NUD* is likely to regulate at least 17 cuticle biosynthetic genes in barley (Duan et al., 2015). The study of Duan et al. (2015) utilized two barley cultivars from very different genetic backgrounds, therefore it is possible that other regulatory elements could be responsible for the observed differences. Although *NUD* may determine the covered or naked phenotype through causing changes in cementing layer development or composition (Taketa et al., 2008; Kakeda et al., 2011), there is no evidence among covered cultivars, that the observed differences in adhesion quality are regulated by *NUD* in the same way.

The compounds that comprise plant cuticles are synthesized in the epidermal cells before transport across the plasma membrane to the cell wall by ATP-binding cassette (ABC) transporters (Kunst and Samuels, 2009; McFarlane et al., 2014), and several reviews of their synthesis can be consulted for greater detail (Shepherd and Griffiths, 2006; Yeats and Rose, 2013; von Wettstein-Knowles, 2016). The outer epidermal cell wall becomes a matrix embedded with the polyester cutin, which in turn is embedded with cuticular waxes, and overlaid with epicuticular waxes which form crystals or plates on the outer cuticle surface. Plant cuticular waxes begin synthesis first in the plastids and are formed from the production of malonyl-CoA by acetyl-CoA carboxylase (ACC). This is followed by elongation to C_16_ or C_18_ acyl chains through addition of C_2_ moieties by the fatty acid synthase (FAS) enzyme complex. The acyl chains are then elongated to very long chain fatty acids (VLCFAs) by the fatty acid elongase (FAE) complex of enzymes, which comprises a 3-ketoacyl-CoA synthase (KCS), a 3-ketoacyl-CoA reductase (KCR), a hydroxyacyl-CoA dehydratase (HCD) and a *trans*-2,3-enoyl-CoA reductase (ECR). Subsequently, the VLCFAs are transported to the endoplasmic reticulum, where they are further modified to other classes of lipids by enzymes either in the decarbonylation pathway (such as alkanes, secondary alcohols, and ketones) or the reductive pathway (such as primary alcohols, fatty acids, and esters). Triterpenoids including sterols are typically minor components of plant cuticular waxes, and are synthesized from acetyl-CoA through the mevalonate pathway to 2,3-oxidosqualene, which is converted to cycloartenol by oxidosqualene cyclases such as CAS1, before being further catalyzed to plant sterols. Some triterpenoids, such as campesterol and β-sitosterol have previously been indicated as being correlated with husk adhesion quality (Brennan et al., 2017a). It has been postulated that the phenotype of husk adhesion is similar to that of organ fusion (Taketa et al., 2008; Duan et al., 2015; Brennan et al., 2017a), in which the organs of plants with mutations in cuticle synthesis genes become adhered. Organ fusion is often associated with changes in the composition of cuticular wax or cutin, and is sometimes correlated with higher than usual cuticular permeability (Lolle et al., 1998; Sinha and Lynch, 1998; Aharoni et al., 2004; Kurdyukov et al., 2006b; Bird et al., 2007; Luo et al., 2007; Rowland et al., 2007; Li et al., 2015; Wang et al., 2015; Jakobson et al., 2016).

This study used different cultivars with a known range of husk adhesion qualities, encompassing extremely good through to poor quality adhesion, to examine the developmental and genetic influences on the quality of husk adhesion. Changes in fresh weight and dimensions of the organs of barley grains were measured during grain filling, and the thickness and composition of the cementing layer were examined, to correlate developmental changes with final husk adhesion quality as measured by grain skinning assessment. Transcriptional changes among cultivars in developing caryopses at two stages of grain development just prior to husk adhesion were measured using a custom barley 60k microarray (Agilent), and significant changes in expression among samples were related to adhesion quality of the cultivars. A secondary objective was to examine environmental and genotype by environment interactions that influence husk adhesion quality. Therefore, to examine potential interactions these cultivars were grown in a glasshouse, both under a misting program known to decrease husk adhesion quality, and also under a control condition where no misting was applied, to examine any correlations between the misting environment and changes in development or gene expression.

## Materials and Methods

### Choice of Cultivars for the Study

Cultivars with different husk adhesion qualities (five good and five poor) were chosen for this study from a panel of 216 cultivars that formed a subset of the IMPROMALT collection which is maintained at the James Hutton Institute (JHI). The panel is listed as “Gh13” (100 cultivars) and “Gh14” (116 cultivars) in Brennan et al. (2017b). Cultivars were grown in four environments followed by skinning assessment as a measure of husk adhesion quality. Glasshouse samples were from Brennan et al. (2017b). There were also samples from three field trials. Trial D2012 was a row and column design with 16 columns of 40 rows in each replicate and a subset of 199 samples from one replicate of the trial were evaluated for skinning. One hundred and seventy-six of the 199 lines from D2012 trial plus 15 new lines were sampled from an unreplicated and randomized plot trial grown at JHI Dundee in 2013 (D2013). One hundred lines that were being evaluated as part of a BBSRC Crop Improvement Research Club project to examine the processability of malting barley (BB/J019593/1), 83 and 17 of which had been in D2012 and D2013, respectively, were grown in an unreplicated trial at SRUC’s Boghall Farm, near Edinburgh (E2013). All three field trials were grown under a malting barley nitrogen management regime and a prophylactic fungicide regime applied to protect the plots from foliar pathogens. When the majority of the plots were ripe, all plots were harvested with a small plot combine and dried samples were cleaned and graded over a 2.5 mm sieve prior to measurement of thousand grain weight with MARVIN (GTA Sensorik Gmbh). Grain samples were assessed for skinning as in Brennan et al. (2017b) where a threshold of husk loss by area was used. Grains with 20% or greater husk loss by area were considered “skinned”, and grains with less than 20% husk loss by area were considered “intact.” GenStat Version 16 was used to build a generalized linear mixed effects model (GLMM) with the logit link function relating the binomial proportions of skinned and intact grains (response variable) to cultivar as the predictor variable. The random term was cultivar nested within the four environments. The cultivars were then ranked in order of predicted proportions of skinned grains. Cultivars with poor husk adhesion were selected from the highest 10% of cultivars in the list by their current commercial relevance. The five cultivars with poor quality husk adhesion were Poker, Braemar, Scandium, Propino, and Concerto. Cultivars with good husk adhesion in the lowest 10% of the list were not often commercially relevant as these were typically older cultivars; skinning is known to be particularly prevalent among recently released cultivars (Brennan et al., 2017b). Therefore cultivars with good adhesion were selected from the lowest 25% of the list, considering the genetic relatedness to the five cultivars chosen for poor quality husk adhesion. The five chosen cultivars with good husk adhesion quality were Henni, Astoria, Prisma, Westminster, and Prestige.

All cultivars were examined for differences in ear and grain development, cementing layer thickness and were assessed for skinning as described below. Experimental work which was more time-consuming was limited to those cultivars which had the most extreme mean skinning phenotype, as cultivars with skinning phenotypes in the middle of the range were not found to be significantly different from the extremes, and these results would not contribute to determining how differences in husk adhesion quality among cultivars is mediated. Therefore for cementing layer composition analyses, only the cultivars Henni, Astoria, Prestige, Concerto, Poker, and Propino were investigated. For the microarray analyses, the same six cultivars were intended for analysis, however, during a laboratory cleanout the harvested Astoria samples were unintentionally discarded, and therefore only Henni, Prestige, Concerto, Poker, and Propino were investigated.

### Plant Growth

The 10 cultivars were chosen to encompass the extremes of husk adhesion qualities. Seeds of these 10 cultivars were sown directly in Levington’s No. 2 compost, with seven seeds being sown in each four-liter pot. After 1 week, any un-germinated seeds were re-sown with fresh seed to maintain a density of seven plants per pot. Pots were spatially replicated four times for each of the two treatments: a misting treatment, and an un-misted control treatment (Brennan et al., 2017b). Within each replicate, cultivar position was randomly organized. Main shoot ears were tagged, and the date of anthesis recorded after visual inspection of the central florets; the date of anthesis was when the anthers were at the top of the floret and were bright yellow. Floral parts were harvested as described below at 5, 10, 15, 25, and approximately 48 days post-anthesis, which corresponded to growth stages 73 (early milk ripe caryopsis), 75 (medium milk-ripe caryopsis), 77 (late milk-ripe caryopsis), 85 (soft dough caryopsis), and 91 (caryopsis difficult to divide). All developmental growth stages (GS) referred to are those of the decimal code described for cereal growth stages by Tottman and Broad (1987). Grain growth and cementing layer analyses were done only on three replicates due to time constraints, whereas microarray analyses and skinning assessments were done on all four replicates. The misting treatment was the same as that described in Brennan et al. (2017b), where 1-min periods of fine mist were sprayed onto plants three times per day at 0900 h, 1300 h, and 1700 h, between anthesis (when approximately half the ears in the experiment had reached anthesis as defined above) and ripening (when approximately half the ears in the experiment reached GS 92, and the caryopses were too hard to dent with a thumbnail). The un-misted control plants did not receive any misting from overhead; all plants were watered directly into pots rather than from above during the duration of the experiment. Once grains were in the final stages of ripening, the misting treatment was ended, allowing grains from the misting and control treatment to complete ripening at similar rates. Depending on the variable being measured, caryopses were harvested from main-shoot spikes at specific developmental stages as described below.

### Grain Development Measurements

Grain growth was measured by sampling fresh husks and caryopses during grain filling at GS 75 and 77, and on the whole grain post-husk adhesion at GS 85 and 91. Husk (palea and lemma separately), caryopsis and grain weights were measured on organs from the central five florets along one side of each spike from each replicate using a Mettler Toledo XP6 microbalance (accurate to ±1 μg), followed by measurements of width and length using calipers (accurate to ±0.05 mm). The harvested ear and grain attributes grain weight, grain number, total floret number (sum of full and “blind” florets) and ear length were measured on ears and grain obtained from 20 ears chosen at random (five ears from each of the four biological replicates), which were then assessed for grain skinning as described below. To determine whether cultivar, treatment or GS had a significant effect on floret development, grain development data was divided into a pre-adhesion phase (GS 75 and 77), and a post-adhesion phase (GS 85 and 91) similar to Brennan et al. (2017a). Individual linear mixed effects models were fitted with each measured attribute as the response variable, cultivar, treatment and GS as fixed effects and replicate number as the random effect. Hierarchical models were compared by ANOVA and non-significant variables were dropped to obtain the final minimally adequate model for each variable. Significant differences among samples were then determined by comparing 95% confidence intervals calculated using the “emmeans” package (Lenth, 2018) in R (R Development Core Team, 2008). To determine whether cultivar, treatment and their interaction had significant effects on the final ear and grain attributes (the 20 ears described above), ANOVA was used, with non-significant variables iteratively dropped as above. This was followed by a *post hoc* Tukey’s HSD test to determine differences among samples for the significant variables influencing that attribute. As non-significant variables were not tested, they are not discussed in the results.

### Cementing Layer Thickness Measurements

Caryopses were sampled from the central three florets of one spike chosen at random from each replicate at GS 75 and 85. Caryopses were harvested from the opposing side of the spike that was sampled for grain growth measurements described above. Segments were excised from the center of the dorsal side of the caryopsis and prepared for electron microscopy as described in Brennan et al. (2017a). Five micrographs of the cementing layer were taken from each replicate, ensuring that each micrograph taken was from a different cell within that replicate. The cementing layer thickness was measured five times per micrograph using ImageJ (Abràmoff et al., 2004) and the mean thickness for each replicate calculated. To determine whether growth stage, or the interaction between treatment and cultivar had a significant effect on the thickness of the cementing layer, a linear model was fitted to the data using the standard deviation of samples within each set of biological replicates as a weighting factor. The variables growth stage, and the interaction between cultivar and treatment were found to have a significant effect on cementing layer thickness, therefore significant differences among all samples were then determined using the “emmeans” package as above.

### Cementing Layer Composition

For the analysis of cementing layer composition, one spike from each replicate was harvested at GS 77, when the caryopses began to feel sticky to touch. The husks were removed from all caryopses on the spike, excluding the top four and bottom two caryopses which were discarded, followed by extraction of the surface lipids by sequentially dipping the caryopses from each ear in 1 ml of dichloromethane (puriss. p.a. grade for GC ≥ 99.9%, Sigma-Aldrich, United Kingdom) for 20 s at room temperature followed by evaporation of the extracts to dryness under N_2_ (British Oxygen Company, 99.995%). Surface lipid extracts were derivatized and analyzed as described in Brennan et al. (2017a). Statistical analysis of differences in compound abundance among samples was done in the same manner as Brennan et al. (2017a). Briefly, for each compound linear mixed effects models were fitted to the data with treatment and the interaction between cultivar and growth stage as fixed effects, and replicate number a random effect. Non-significant terms were iteratively dropped from the models starting from the higher-order terms, to obtain a minimal model. The “emmeans” package was then used to determine which samples differed in abundance of individual compounds as above. As non-significant variables were not included in *post hoc* pairwise comparisons, they are not discussed in the results.

### Microarray Analysis

A smaller subset of cultivars with phenotypic extremes comprising the resistant cultivars Henni and Prestige, and the susceptible cultivars Concerto, Poker, and Propino were chosen for comparative transcriptomic analysis at growth stages 73 and 75. The smaller subset was chosen because time and resources were limited. For each of the four biological replicates per cultivar and growth stage, the three central caryopses along one side of each spike were dissected and frozen immediately in liquid nitrogen. These growth stages were chosen to include the physiological stage before, and during, peak expression of *NUD*, which was determined by a pilot study using qRT-PCR to measure *NUD* expression at GSs 71, 73, and 75 on the cultivar Concerto (data not shown). The caryopses were ground in liquid nitrogen using a pestle and mortar, followed by extraction of total RNA using TriReagent (Sigma-Aldrich) according to the manufacturer’s instructions. RNA integrity was assessed using a Bioanalyzer 2100 (Agilent Technologies) with RNA 6000 Nano Reagents (Agilent Technologies) and RNA quantified using spectrophotometry. Microarray processing was performed using a custom-designed barley Agilent Microarray (A-MEXP-2357 ^1^), as described in Comadira et al. (2015), representing transcripts derived from *c*. 61,000 barley gene models (International Barley Genome Sequencing Consortium et al., 2012). Microarray processing was performed according to the One-Color Microarray-Based Gene Expression Analysis protocol (v 6.5; Agilent Technologies). Data were extracted using Feature Extraction software (v 10.7.3.1; Agilent Technologies) with default settings. Full microarray experimental design and data can be found at E-MTAB-7429 (see text footnote 1). Raw data files were imported into GeneSpring GX (v 7.3; Agilent Technologies) software. Data were normalized using default Agilent Feature Extraction one-color settings (raw intensity values were set to a minimum of 5; values were normalized to the median of all probe signals in each array; values for each probe were normalized to its median value across all arrays) and filtered to remove inconsistent probe data flagged as absent in more than one replicate per sample. Normalized data were exported for subsequent statistical analyses (see below). In total, 38,538 probes passed QC filtering and were further analyzed for significant differences in expression. For each probe that passed the filter, a linear model was used to test whether treatment, cultivar, growth stage and the interaction between cultivar and growth stage had a significant effect on expression of the associated gene. Benjamini-Hochberg correction was used to control the false discovery rate at 5%. Neither treatment nor the interaction between cultivar and growth stage had a significant effect on gene expression detected by any probe. Both cultivar and growth stage significantly affected expression of 2,015 transcripts, growth stage significantly altered affected expression of 11,771 transcripts, and cultivar significantly altered expression of 1,814 transcripts. These probes and associated genes with significant differential expression are listed in Supplementary Table S1. For these probes, analysis of variance (ANOVA) was followed by a *post hoc* Tukey’s HSD test with the significant variables to determine which samples had significantly different expression levels from each other. There were 23,426 probes where neither growth stage nor cultivar significantly affected associated gene expression levels.

### Skinning Phenotyping for Adhesion Quality

At harvest ripeness, 20 healthy ears of each subset cultivar were harvested for skinning assessments as described above. Ears were first hand-threshed to produce clean grain by snapping off the awns and carefully removing the grains from the rachis so as not to cause husk damage, followed by threshing in a Wintersteiger LD 180 laboratory thresher (Wintersteiger AG, Ried, Austria) for 20 s to cause skinning. Each ear was then assessed for grain skinning as above. To determine whether cultivar type or the misting treatment influenced skinning severity, a generalized linear mixed effects model was fitted using the package “lme4” (Bates et al., 2015) in R (R Development Core Team, 2008). The proportion of skinned grains was the response variable, with cultivar, treatment and their interaction as fixed effects, and replicate number as the random effect. Terms were sequentially dropped from the full model and hierarchical models compared using analysis of variance (ANOVA) to determine which variables were significant. The interaction between cultivar and treatment was found to be significant (*p* < 0.0001), therefore differences among all cultivars and treatments were determined by comparing 95% confidence intervals using the package “emmeans” as above.

## Results

### Effect of Misting and Cultivar on Skinning

**FIGURE 1:**
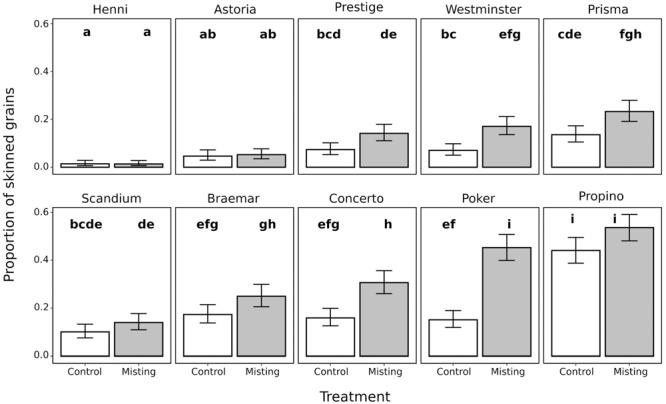
Proportion of skinned grains in a panel of 10 cultivars encompassing a range of husk adhesion qualities, which were grown under a misting treatment or a non-misted control treatment. The misting treatment typically had a negative effect on the quality of husk adhesion. Samples which do not share a letter are significantly different from each other. Values are the fitted mean proportion of skinned grains ±95% confidence intervals.

The misting treatment caused significantly lower quality husk adhesion and therefore higher skinning than the control treatment in four of the 10 cultivars assessed (Figure 1). Within the control treatment, Henni had lower skinning than all other cultivars examined, and Astoria had lower skinning than all of the cultivars chosen for historically poor quality adhesion except for Scandium. The other three cultivars chosen for historically high quality adhesion were Prestige, Westminster, and Prisma which all had skinning levels which were not significantly different from the poor quality cultivars, except for Propino which had significantly higher skinning than all cultivars. Within the misting treatment, a similar pattern was observed except that both Henni and Astoria had significantly lower skinning than all other cultivars. Cultivars which had extremely good or extremely poor husk adhesion were not affected by the misting treatment, therefore there was a significant interaction between cultivar and treatment (*p* < 0.001), no cultivars had lower husk adhesion in the misting treatment than in the control treatment. In the cultivars which have high quality husk adhesion, Henni, Astoria, and Prestige, there was no significant increase in skinning in grains from the misting treatment compared with the control treatment. Among cultivars with moderate quality husk adhesion, Westminster and Prisma had significantly higher skinning in grains from misted plants than in grains from control plants. Conversely, the misting treatment had no effect on Scandium and Braemar. Concerto and Poker, cultivars with poor quality husk adhesion, had significantly higher skinning in grains from the misting environment than in the control grains. The misting treatment had no effect on skinning in Propino, which has the lowest quality husk adhesion, and had extremely high skinning levels in grains from both the misting and control treatments. For comparison with field data, Propino had 46% skinned grain in the D2012 trial, which showed the highest levels of skinning of the three field experiments that we studied. By contrast, Henni had 5% skinned grain in the D2012 trial, therefore the results that we have observed are consistent with the field observations. These results indicate that there is a large genotype by environment interaction that determines the final quality of husk adhesion for malting cultivars, and that this is particularly the case for cultivars which do not have extremely good, or extremely poor, husk adhesion.

### Ear Physiology

**FIGURE 2:**
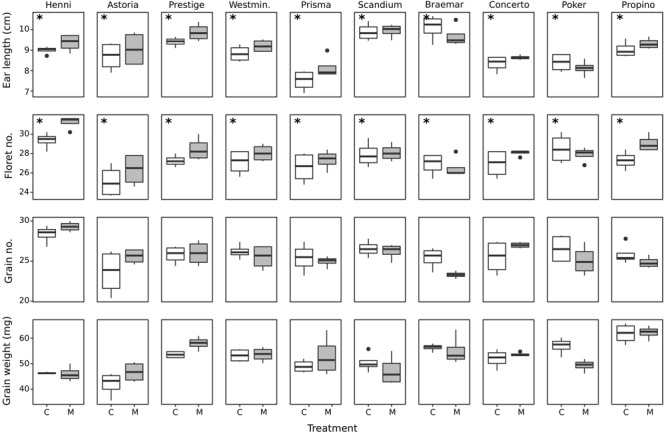
Boxplots of ear and grain measurements made at harvest ripeness for the 10 cultivars grown under control and misting treatments. Cultivars are arranged from best to worst quality husk adhesion. Cultivars which had a significant differences between treatments are indicated with an asterisk. C, control treatment; M, misting treatment.

The ear and grain attributes measured at harvest are shown in Figure 2. For ear length and floret number, both misting and cultivar had a significant effect, with no significant interaction. There was no clear trend that cultivars with good or poor quality husk adhesion consistently had long or short ears, or high or low floret number, grain number or grain weight. Misting significantly increased mean ear length by 0.2 cm over the control treatment (*p* < 0.04). Scandium and Braemar had the longest ear length, with significantly longer ears than Astoria, Westminster, Prisma, Concerto, and Poker. Prisma had significantly shorter ears than all cultivars except for Concerto and Poker, likely to be a result of reduced rachis internode length in this cultivar. Misted plants had significantly higher floret number by a mean of 0.8 florets per ear (*p* < 0.003). Henni had the highest number of florets, with a significantly higher number of florets than all other cultivars. Henni also had significantly higher grain number than all other cultivars, whereas no other cultivars had significantly different grain numbers from the others. Astoria had the lowest number of florets, which was significantly lower than Poker, Prestige, Propino, and Scandium. There were no other differences in floret number among cultivars. There was a significant interaction between cultivar and treatment for grain weight (*p* < 0.05) largely due to Astoria control grains having significantly lower weight than grains from most cultivars following misting treatment. Within each cultivar, there were no significant differences in grain weight between the misting and control treatments. Pairwise comparisons between cultivars grown under different treatments were not of interest in this study. Within the control treatment, Astoria had significantly lower grain weight than Prestige, Westminster, Scandium, Braemar, Poker and Propino; and Propino had significantly higher grain weight than Prisma.

### Development of Grain Components

The measurements of length, width, and weight from developing grain organs at GS 75, 77, 85, and 91 are given for each cultivar and treatment in Supplementary Table S2. Post-adhesion measures taken at GS 85 and 91 necessarily include the husk organs as part of the caryopsis measures, as the husk could not be removed without causing damage. No clear trends emerged between cultivars with high or low quality husk adhesion, and either particularly large or small grain components, and the treatment rarely had an effect on grain development. Cultivar had a significant influence on all pre-adhesion measures of organ development (*p* < 0.001 for all). *Post hoc* tests showed there were few significant differences in caryopsis length among cultivars, but Propino had significantly wider caryopses than all cultivars except Westminster. At both GS 77 and 75, for both treatments, Henni and Astoria typically had significantly lower caryopsis weight than all other cultivars, whereas Propino and Westminster had significantly higher caryopsis weight than most of the other cultivars. Propino and Prestige had significantly longer lemmas than all other cultivars, and Propino had significantly wider lemmas than all cultivars except Concerto and Poker. Propino, alongside Prestige, also had higher lemma weight than all other cultivars. Concerto had wider paleas than Astoria, with few other cultivar differences significant in *post hoc* tests. Correspondingly, Astoria also had significantly lower palea weight than all other cultivars. Caryopsis length (*p* < 0.7), lemma length (*p* > 0.2) and palea length (p < 0.9) did not change between GS 75 and 77, but caryopsis width was always significantly wider at GS 77 than GS 75 (*p* < 0.001). Lemma width increased at GS 77 compared to GS 75 (*p* < 0.001), but conversely, palea width decreased (*p* < 0.001). During the pre-adhesion growth phases, treatment was found to have a significant effect on caryopsis length (*p* < 0.001) and caryopsis weight (*p* < 0.009), but not width (*p* < 0.4), however, there were no significant differences between treatments found in *post hoc* tests.

In the post-adhesion growth stages, caryopses were significantly longer at GS 85 than 91 (*p* < 0.001), and wider at GS 85 (*p* < 0.001), reflecting the loss of grain water that occurs between GS 85 and 91. Caryopsis weight was also significantly lower at GS 91 than 85 (*p* < 0.001), accordingly. Although treatment was a significant factor influencing caryopsis width (*p* < 0.004) and weight (*p* < 0.003), there were no significant differences among samples identified by *post hoc* tests. However, the misting treatment significantly increased caryopsis length for cultivars Astoria, Prestige, Westminster, Concerto, and Poker at GS 85, and for Prisma only at GS 91. At both GS 85 and 91, Astoria, Westminster and Concerto had significantly shorter caryopses than Propino, and also than Prisma with the exception of the misting treatment at GS 85. Although Astoria also had significantly less wide caryopses compared with Propino at all growth stages and treatments, Concerto had significantly wider caryopses than most other cultivars indicating a difference in grain morphology between these cultivars, with Concerto having rounder grains than Astoria. Astoria and Prisma had significantly lower caryopsis weight than Braemar and Propino for all treatments and growth stages.

### Cementing Layer Development and Thickness

**FIGURE 3:**
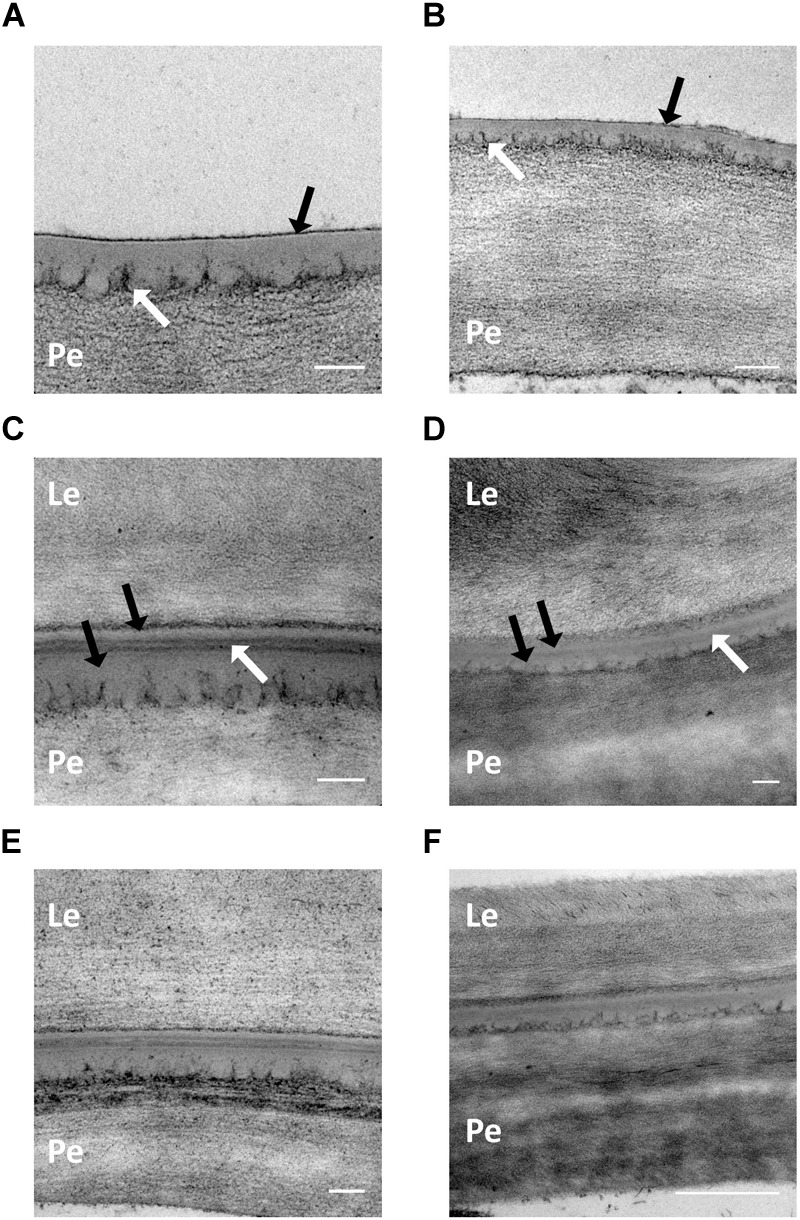
The cementing layer under formation and post-adhesion. **(A)** Henni GS 75, control treatment, scale = 0.1 μm. **(B)** Propino GS 75, control treatment, scale = 0.2 μm. There is an electron-dense material forming on the outer surface of the pericarp cuticle (black arrows) in both cultivars at GS 75. The cell-wall pectic polysaccharides can be seen extending into the cuticular matrix (white arrows). **(C)** Henni GS 85, control treatment, scale = 0.1 μm. **(D)** Propino GS 85, control treatment, scale = 0.1 μm. The cementing layer of both cultivars comprises the pericarp and husk (lemma) cuticles (black arrows), with an electron-dense cementing material in between (white arrows). Lamellations within the cementing material can be seen in **(C)**. **(E)** Henni GS 85, misting treatment, scale = 0.1 μm. **(F)** Propino GS 85, misting treatment, scale = 0.5 μm. The morphology of the cementing material is not different from samples grown under the misting treatment. Pe, pericarp cell wall; Le, Lemma cell wall.

Development of the cementing layer was examined at GS 75 (pre-adhesion) and GS 85 (post-adhesion) using transmission electron microscopy. A selection of micrographs from samples of Henni and Propino, the cultivars with extremes of husk adhesion qualities, are shown in Figure 3. At GS 75, both Henni (Figure 3A) and Propino (Figure 3B) have a thin electron-dense material developing on the outer surface of the pericarp cuticle (black arrows), which is likely to be the beginning of the development of the cementing material. The pectic polysaccharides of the cell wall can be seen as dark tendrils extending into the cell-wall-cuticle matrix (white arrows) (Jeffree, 2008). At GS 75, there is no evidence of an irregular interface containing globules of cuticular material between the cell wall and cuticle, indicating that production of the cementing material is proceeding slowly at this growth stage. Post-adhesion, at GS 85, the cementing layer has fully developed. Examples of Henni (Figure 3C) and Propino (Figure 3D) grown under the control treatment show that the cementing layer comprises the cuticles of the pericarp and the husk which appear electron translucent (black arrows), with the electron-dense cementing material filling the space between the two cuticles (white arrows). The cementing material can be either lamellated (Figure 3C) or amorphous (Figure 3D), both types occurred in all cultivars, and within all replicates. The misting treatment did not cause any clear structural changes in either Henni (Figure 3E) or Propino (Figure 3F), or the other cultivars examined.

**FIGURE 4:**
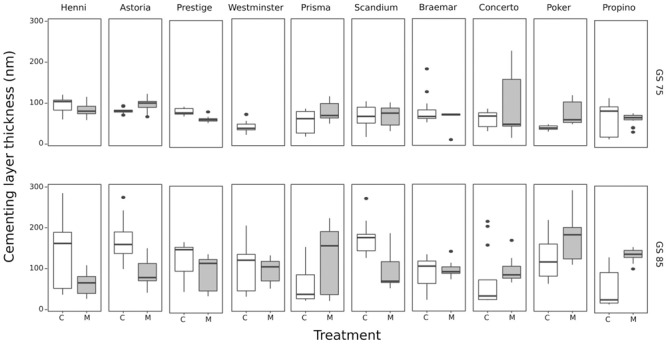
Development of cementing layer thickness at GS 75 and GS 85 in 10 cultivars grown under control and misting treatments. At GS 85, the mean thickness was greater due to the measurement comprising the husk and caryopsis cuticles, as well as the cementing material. Cultivars are arranged from best to worst quality husk adhesion. There is no clear correlation between husk adhesion quality and cultivar or the misting treatment. C, control treatment; M, misting treatment. Here, to better display the variation, *n* = 15 from three biological replicates each of which had the cementing layer thickness measured on five different cells.

The thickness of the cuticle on the caryopses at GS 75, and thickness of the entire cementing layer at GS 85, is shown for each cultivar grown in the control and misting environments in Figure 4. The Westminster GS 75 samples lost some cell layers during processing, leaving insufficient samples for statistical analysis and so were not included. Growth stage had a significant effect on the thickness of the caryopsis cuticle (*p* < 0.001) which increased from a mean of 70.34 nm at GS 75 before the cementing layer truly began to develop, to a mean of 109.29 nm at GS 85 after the cementing layer had developed and adhesion had taken place. At GS 85 the cementing layer measurement comprises the inner cuticle of the lemma, the cuticle of the caryopsis and the electron-dense cementing material between these two cuticles. There was a significant interaction between cultivar and treatment (*p* < 0.01), caused by samples from the misting treatment sometimes having increased mean cuticular thickness than the control samples in some cultivars, and thinner mean cuticle thickness in others. However, comparison of 95% confidence intervals indicated that none of the samples were significantly different from each other. At GS 85, 11 of the 20 samples (10 cultivars × 2 treatments) had a sufficient number of replicates where the boundaries between the cuticles of the husk, and the electron-dense material were distinct enough to measure that the effects of cultivar and treatment could be tested. The mean thickness was 40 nm, and neither treatment (*p* < 0.2) nor cultivar (*p* < 0.5) had a significant effect. It is possible that differences might have been significant if a greater number of the cultivars could have been tested, although the variability observed among measurements was not much less compared with the variability among cultivars, making this unlikely.

### Changes in Cuticular Lipid Composition

Changes in the composition of solvent-extractible lipids was measured at GS 75 (before the caryopsis feels sticky), and at GS 77 (when the caryopsis feels sticky, and is near maximum volume). For this analysis, six cultivars were investigated which were sufficient to cover the full range of the skinning phenotype: Henni, Astoria, Prestige, Concerto, Poker, and Propino. A total of 116 compounds were identified in the caryopsis surface lipid extracts. These compounds were the same as those listed in Brennan et al. (2017a), except that not all esters were represented in sufficient quantity for quantification and two unknown compounds “5” and “6” were not present. The compounds were quantified using the same ions as listed in Brennan et al. (2017a). The compounds identified included members of the following classes: alcohols, alkanes, alkenes, alkyl resorcinols, alkyl esters, fatty acids, ketones, and triterpenes, and four unidentified compounds. Of these, 44 had significant changes in abundance depending on treatment, cultivar and growth stage, or the interaction with cultivar and growth stage. The fitted means, standard errors and significant differences of the 44 compounds are given in Supplementary Table S3, depending on the significant variables in the final models. Where variables did not have a significant effect on the abundance of that compound, they were not considered.

The alkanes tricosane (C_23_) and tetracosane (C_24_) were the only two compounds in which treatment, and the interaction between cultivar and growth stage were all significant. Both of these compounds were always significantly higher in abundance at GS 75 than GS 77, expect for in the cultivar Poker in which their abundance was highest at GS 77 for both treatments. Although the effect of treatment was significant, *post hoc* tests showed no significant differences among samples of the same cultivar and growth stage. The alcohols tetracosanol (C_24_) and pentacosanol (C_25_) also had treatment as a significant effect, although there were no significant differences in abundance within each cultivar and growth stage. These alcohols were also significantly higher in abundance at GS 75 than at GS 77. At GS 77, Henni and Astoria both had significantly higher proportions of tetracosanol than Poker and Propino.

A total of 39 compounds were not affected in abundance by treatment type, but had a significant interaction between cultivar and growth stage. Within the triterpenoids, campesterol, a compound previously correlated with good quality husk adhesion only significantly increased in abundance from GS 75 to GS 77 within the cultivar Henni. At GS 77, Henni had significantly higher abundance of campesterol than all other cultivars at this growth stage. Conversely, cholesta-3,5-diene was present in significantly higher proportions in Astoria at GS 75 than GS 77. In general, the abundance of alkanes and alkenes was higher at GS 75 than 77, but this was not always significant. In the cultivar Henni, only pentacosane was significantly more abundant at GS 75. In Prestige, heneicosane (C_21_), heptacosene (C_27_), nonacosene (C_29_) and hentriacontene (C_31_) were significantly more abundant at GS 75. Henni had a higher abundance of hexacosanol (C_26_) at GS 75 than GS 77, whereas Astoria had a higher abundance of tricosanol (C_23_) at GS 75 than 77; Propino had significantly higher abundance of both compounds at GS 75 than 77. There were no significant differences in abundance of any fatty acids between growth stages within cultivar. Among cultivars, the abundance of fatty acids tended to decrease from cultivars with good adhesion to those with poor adhesion, particularly at GS 77. Few differences in abundance were significant, however, Henni had higher abundance of tetradecanoic acid (C_14_) and 9-octadecanoic acid (C_18_) at GS 77 than Concerto and Propino at this growth stage.

### Transcriptional Changes Associated With Husk Adhesion

**FIGURE 5:**
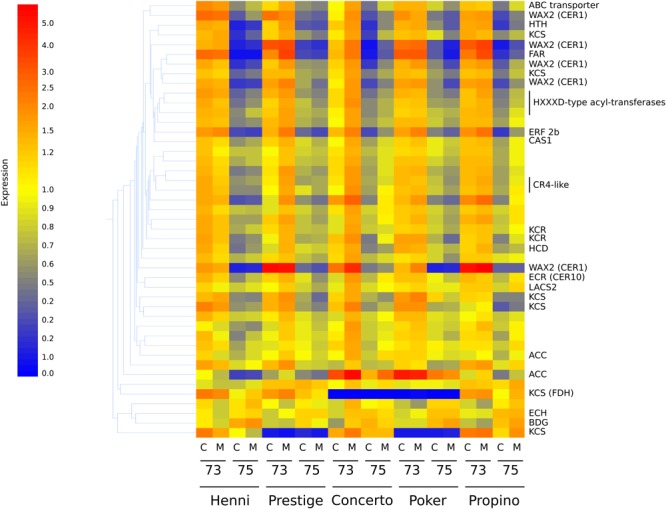
Heatmap showing the relative expression of 45 transcripts corresponding to cuticle synthesis or regulatory genes. Associated genes with high relative expression are represented by the color red, whereas genes with low relative expression are represented by the color blue, through yellow as intermediate relative expression. Gene expression corresponding to acetyl-CoA carboxylase (ACC), important early in fatty acid biosynthesis, is significantly higher in samples at GS 73. Lower expression, corresponding to many lipid biosynthetic genes at GS 75 is common, including genes encoding substituents of the fatty-acyl elongase enzyme complex such as 3-ketoacyl-CoA synthases (KCS), hydroxyacyl-CoA dehydratase (HCD), and to a lesser extent 3-ketoacyl-CoA reductases (KCR), a *trans*-2,3-enoyl-CoA reductase (ECR) and 3-hydroxyacyl-CoA dehydratases. The putative WAX2 fatty-acyl hydroxylases are thought to catalyze steps in the decarbonylation pathway. C, control treatment; M, misting treatment; 73, growth stage 73, 75, growth stage 75.

Differences in transcript abundance are presented below in accordance with the highest-matching gene homolog. The misting treatment did not have a significant influence on transcript abundance, indicating that the effect of misting on husk adhesion quality is likely to be physical in nature rather than a physiological response of the plant to the treatment. There were 11,771 probes which detected significantly altered transcript abundance between GS 73 and GS 75, but which did not have significant differences among cultivars. On the entire array, there are 83 probes which are known to correspond to sequences of genes for biosynthesis of cuticular compounds, regulation of cuticle biosynthesis or transport of cuticular products. Of these, 45 indicated significantly different transcript abundance among cultivar (one probe), growth stage (40 probes) or both (4 probes) (Supplementary Table S4). The relative expression of these probes is shown in the heatmap in Figure 5, and clearly demonstrates that treatment type had little or no effect on each cultivar.

Almost all of these 45 transcripts were significantly downregulated from GS 73 to 75. The only exceptions which were upregulated were those corresponding to a gene for *Echidna* (*ECH*), which is required for the secretion of wax from epidermal cells (McFarlane et al., 2014); *Bodyguard* (*BDG*), encoding an α/β-hydrolase localized to the outer epidermal cell wall and thought to be involved in determining cuticle structure and polymerization of carboxylic esters (Kurdyukov et al., 2006a; Jakobson et al., 2016); and a MYB96 transcription factor, mutants of which have reduced stem wax (Seo et al., 2011; Guo et al., 2013). The transcript corresponding to the NUD ethylene responsive transcription factor was not significantly differently expressed between growth stages or among cultivars. However, transcripts corresponding to other genes known to regulate cuticle biosynthesis had significantly lower expression at GS 75 than at GS 73. These genes included those encoding the AP2-domain containing ethylene responsive factor GL15 involved in epidermal cell identity (Evans et al., 1994; Moose and Sisco, 1994, 1996), another AP2-domain ethylene responsive transcription factor WXP1 (Zhang et al., 2005), and also CR4 receptor kinase, MYB transcription factors and homeobox-domain-containing transcription factors. The transcript corresponding to the CER9 ubiquitin protein ligase which elevates both very long-chain fatty acid synthesis and cutin synthesis (Lü et al., 2012), and which is more highly expressed in covered barley compared to naked barley (Duan et al., 2015), was down-regulated in GS 75 compared to GS 73 in our study.

As well as regulatory genes, transcripts corresponding to cuticular lipid biosynthesis genes also had significantly altered abundance between GS 73 and GS 75, but not among cultivars. At GS 73, expression of ACC which is required for production of the fatty acyl chain precursor malonyl-CoA, is significantly higher than at GS 75. Similarly, transcripts corresponding to genes that are part of the fatty acyl elongase complex had higher expression at GS 73 than 75. This included transcripts for KCS, KCR, a HCD, and a *trans*-2,3-enoyl-CoA reductase (ECR). Several other transcripts for putative WAX2 fatty acyl hydroxylases, which are thought to be involved in the production of secondary alcohols and ketones via the decarbonylation pathway (Rowland et al., 2007) were found. A gene representing choline dehydrogenase *Hothead* (HTH), implicated in cutin biosynthesis (Kurdyukov et al., 2006b; Xu et al., 2017), had significantly lower expression at GS 75. Transcripts encoding CAS1 cycloartenol synthase (Gas-Pascual et al., 2014), an enzyme required for cyclization of 2,3-oxidosqualene to cycloartenol in plant sterol biosynthesis, also had significantly lower abundance at GS 75 than GS 73. The same expression pattern was seen for transcripts of a gene for a putative sterol-4-methyl oxidase, an enzyme which converts cycloartenol to phytosterols such as campesterol and cholesta-3,5-diene. Transcripts corresponding to putative GDSL esterase/lipases were also more abundant at GS 75 than 73; a barley GDSL eterase/lipase at the *cer-zv* locus has previously been associated with poor husk adhesion (Li et al., 2017), and another in *Arabidopsis* is associated with organ fusion (Takahashi et al., 2010). The *cer-zv* mutant phenotype, however, is more similar to naked barley, where adhesion never takes place, than to grain skinning.

Cultivar alone affected expression of 1,814 transcripts, of which only one was known to correspond to a gene involved in cuticle biosynthesis, long-chain acyl-CoA synthase 2 (*LACS2*), encoding a fatty-acyl activation enzyme with a role in cutin biosynthesis (Schnurr et al., 2004; Lü et al., 2009). This had significantly lower expression in Henni and Poker, than in Prestige or Propino (Supplementary Table S4). Expression of 2,015 transcripts were significantly influenced by both cultivar and growth stage. Of these, four were known to correspond to genes involved in cuticular lipid biosynthesis or regulation (Supplementary Table S4). These encoded three 3-ketoacyl CoA synthases, one of which corresponds to the Fiddlehead (FDH) 3-ketoacyl CoA synthase, mutants of which have reduced wax loads and organ fusion phenotypes (Lolle et al., 1997; Yephremov et al., 1999; Pruitt et al., 2000). These were significantly downregulated in GS 75 compared with GS 73. The fourth transcript corresponded to an ACC, an enzyme necessary for the production of malonyl CoA substrates for fatty acid biosynthesis; this was also significantly downregulated in GS 75. Among cultivars, two of the three 3-ketoacyl CoA synthases (including *FDH*) had significantly lower expression in Poker (poor adhesion) compared to Henni (good adhesion), but also compared to Propino (worst adhesion). The remaining 3-ketoacyl CoA synthases, and the acetyl-CoA-carboxylase, had the opposite expression pattern, being significantly more highly expressed in Poker compared to Henni and Propino. Although they had opposing extremes of husk adhesion quality, Henni and Propino did not have any differences in expression level of these three genes or *LACS2*.

## Discussion

The significant interaction between cultivar and the misting treatment confirms that the quality of husk adhesion in grains of malting barley is under both genotypic and environmental control. Previously, Concerto was shown to have significantly higher levels of grain skinning and therefore poor husk adhesion when grown under the misting treatment (Brennan et al., 2017b). Another field study simulating rainfall events during grain filling in a less controlled fashion also resulted in poor quality husk adhesion compared to plots without additional simulated rainfall (Froment and South, 2003). The study of Froment and South (2003) was done after observations that a year with high rainfall during grain filling resulted in high levels of grain skinning, and it was hoped that inclusion of the misting treatment could aid our understanding of some of the environmental and genotype by environment variation in husk adhesion quality. Although the misting treatment reduced the quality of husk adhesion in some cultivars, it is still not clear exactly how this effect was mediated, or why some cultivars were not affected. That there were no significant changes in the solvent-extractible surface lipids of the caryopses in response to treatment, nor any changes in cementing layer thickness, nor response to treatment observed in the microarray results, the data suggest that the effect of misting or rainfall on the quality of husk adhesion is likely to be a physical process, where water or high humidity inside the florets or on the caryopsis surface disrupts adhesion, in much the same way that adhesives do not easily stick to wet surfaces. If water ingress into florets plays a role in husk adhesion quality, some of the genotypic differences in observed husk adhesion quality might be confounded by differing floret and ear morphologies among cultivars allowing different levels of moisture ingress. However, it was noted (data not shown) that Henni (best adhesion) has particularly tightly closed florets, whereas Propino (worst adhesion) has very loose and open floral parts, but neither had a significant treatment effect on skinning. There was little evidence that skinning differences were due to incompatible husk-caryopsis dimensions, in agreement with the observations of Brennan et al. (2017a). Misting resulted in longer caryopses for five cultivars at GS 85, but only one by harvest ripeness, giving some indication that misting had an influence on ripening behavior. It is possible that the duration of the ripening process could influence grain morphology during husk-caryopsis contact and adhesion, altering the stresses within the cementing layer and between the floral organs, thereby changing adhesion quality. The observed increase in florets under the misting treatment was less than one per ear, and could possibly be due to the misting treatment having to be applied on the sides of the glasshouse where the apparatus was set up (detailed in Brennan et al., 2017b), rather than being able to be randomly interspersed with the control plants. It is possible that the glasshouse sides had different light qualities than the center, and resulted in slightly more vigorous growth during ear development. In the previous study, no significant difference in floret number between the misting and control treatment was observed (Brennan et al., 2017b), and in the current study the increase in florets did not contribute to an increase in final grain number, suggesting that the extra blind florets may have been produced too late for development into viable carpels.

There were no clear differences in the structure of the cementing layer among cultivars, by GS 85 all samples had examples of cementing layers with and without lamellations in the cementing material, and with or without distinctly delineated boundaries between the cementing material and the cuticles of the husk and caryopsis. The thickness of the cementing layer, comprising the cuticles of the pericarp and husk, and the cementing material in between, can be highly variable depending on the anatomy of the grain. Differing morphologies near trichomes on the caryopsis surface, or vascular bundles, can cause some separation between the husk and pericarp which is filled, often incompletely, with cementing material (Gaines et al., 1985; Brennan et al., 2017a). The same partial filling was observed in the present study, although care was taken to measure the cementing layer at regions distant from these areas prone to separation. Regardless, the lack of correlation between cementing layer or cementing material thickness and the quality of husk adhesion indicates that more cementing material does not confer better quality adhesion.

When observed by transmission electron microscopy, the phenotype of husk adhesion has similarities to the phenotype of organ fusion observed in floral parts, leaves and stems of some plants with mutations in cuticular lipid biosynthesis, transport or regulatory genes (Lolle et al., 1992; Sinha and Lynch, 1998; Kurdyukov et al., 2006a,b; Bird et al., 2007; Luo et al., 2007) or transgenic plants expressing fungal or plant cutinases (Sieber et al., 2000; Takahashi et al., 2010). In Arabidopsis *fiddlehead* (*fdh*) mutants which display organ fusion, an electron-dense layer is reported to be present between cuticles of adjacent organs, which becomes less distinct during later development (Lolle et al., 1992). The maize *adherent 1* (*adh1*) mutant has a fusion zone which is also very similar to husk adhesion, with a clearly defined cuticular layer between organs (Sinha and Lynch, 1998). Others have an intermediary cuticular layer which is discontinuous between fused cell walls, including *bodyguard* (*bdg*) (Kurdyukov et al., 2006a), *hothead* (*hth*) (Kurdyukov et al., 2006b), *atwbc11* (Luo et al., 2007) and Arabidopsis expressing a fungal cutinase (Sieber et al., 2000). It is not always clear whether the cuticular layer is discontinuous in these mutants before fusion takes place and subsequently disintegrates, or whether fusion takes place because the cuticular layer is discontinuous in the first place as it is in *hth* (Kurdyukov et al., 2006b). In our study at GS 85 the cementing layer was always present, never being re-integrated into the cell walls, although the electron-dense cementing material was not always distinctly delineated between the two cuticles. However, grains at harvest-ripeness were not examined by electron microscopy in our study, and we cannot rule out that later in grain ripening the cell walls become fused with disintegration of the intermediary cuticles or the cementing material. When the husk is removed at GS 77, during the adhesion phase, the cementing layer can separate from the pericarp cell wall entirely, or separation between the cementing material and the pericarp cuticle occurs (Brennan et al., 2017a, Additional file 3). Expression of a GDSL esterase/lipase in Arabidopsis, cuticle destructing factor 1 (*cdef1*), resulted in a similar phenotype where the cuticle appears to be a bilayer which can separate from itself or the underlying cell wall, causing a discontinuous cuticle (Takahashi et al., 2010). Many of the mutants that display organ fusion phenotypes also have high cuticular permeability reported as measured by toluidine blue O staining (Tanaka et al., 2004) or fresh weight loss over time. This permeability is often postulated to be due to changes in cuticular composition. However, if the cuticle is discontinuous, both of these methods would result in increased cuticle permeability being reported, without the lipidic cuticle itself necessarily having higher permeability. Rather, the staining would be due to exposed cell walls, and weight loss would be due to a lack of cuticle which would otherwise prevent or reduce transpiration. Covered barley caryopses are dyed by Sudan black B after cementing material development (Taketa et al., 2008) despite the fact that there is a continuous cuticle on the caryopsis in addition to the cementing material, whereas naked barley, which only has a thin cuticle (Brennan et al., 2017a) lacking solvent-extractible cuticular waxes (data not shown) does not dye. This indicates that either a compound specific to the cementing material binds the Sudan black B dye in covered barleys, or that development of the cementing layer renders the entire cuticle permeable to the dye.

Changes in the composition of solvent-extractible surface lipids previously reported from caryopses of the cultivar Concerto during the development of husk adhesion were subtle, but typically involved a decrease in proportions of alkanes from GS 75 to GS 77, and increased proportions of some fatty acids from GS 75 to GS 77 (Brennan et al., 2017a). Compounds that were correlated with the quality of husk adhesion in that study included the sterols campesterol and β-sitosterol. In the present study, compositional changes were also subtle, which reflects that comparisons were being made among modern barley cultivars with restricted biological variation in phenotypes rather than among mutants with extreme phenotypes for example. Here, the proportions of alkanes also tended to be higher at GS 75 than GS 77, and cultivars with better quality adhesion tended to have higher proportions of fatty acids. Additionally, the sterols campesterol and cholesta-3,5-diene were present in higher proportions at GS 77 in the cultivars Henni and Astoria, respectively, which both have good quality husk adhesion. Even between mutants that display organ fusion and their wild-type counterparts, changes in cuticular composition are typically subtle. The *wax2* organ fusion mutant was reported to have no changes in cutin load or composition, but a shift in primary alcohols from C_26_ and C_28_ to those of C_30_ (Rowland et al., 2007), and reduced proportions of alkanes, aldehydes, secondary alcohols, ketones and fatty acids (Wang et al., 2015). Conversely, the *atwbc11* mutant displaying organ fusion had higher proportions of secondary alcohols and ketones, and a reduced cutin load (Luo et al., 2007). The *bdg* mutant also had reduced loads of cutin and cutin monomers (Jakobson et al., 2016). If the husk adhesion process is mediated through compositional changes of the cementing layer in a similar process to organ fusion, it appears that minor changes in composition are sufficient to cause adhesion. Cuticular permeability is associated with changes in cuticle composition. As well as compositional changes to the cuticles, the mutant *atwbc11* and *bdg* also have increased cuticular permeability reported (Luo et al., 2007; Jakobson et al., 2016). The similarities between organ fusion and cuticular permeability has led to speculation that husk adhesion may be mediated through changes in cuticular permeability (Taketa et al., 2008; Duan et al., 2015; Brennan et al., 2017a). Sterols of caryopsis cuticles were correlated with adhesion quality in the present study and also that of Brennan et al. (2017a), with differential expression of *CAS1* and a sterol-4-methyl oxidase in the present study further confirming a role of sterols in husk adhesion. Indeed, successful prediction of adhesion quality using Raman microspectroscopy with principal component regression exploited signals from aromatic ring-containing compounds in cementing layer extracts (Brennan et al., 2019). In Arabidopsis, ectopic expression of lupeol synthase, another oxidosqualene cyclase, increased cuticle permeability (Buschhaus and Jetter, 2012), although organ fusion was not reported. In tomato (*Lycopersicon esculentum*) fruit, higher proportions of alkanes have also been linked to low cuticle permeability (Vogg et al., 2004), although not organ fusion. Together, these results suggest that increased proportions of sterols or other triterpenoids, and reduced proportions of alkanes, observed in caryopses at GS 77, potentially contribute to adhesion through altering cuticle permeability. Cuticle permeability of the caryopses was not measured in this study, and would be need to be timed very carefully to coincide with the developmental stage when the cementing material has been produced, but before removal of the husk causes the type of damage to the cuticle surface as seen in Brennan et al. (2017a). If microscopic tears in the cuticle surface were caused by husk removal after even a small amount of adhesion, this would result in high permeability being observed, and therefore correlated with husk adhesion quality, without necessarily being a true measure of cuticle permeability.

Although cultivar-specific expression of some *KCS* genes including *FDH*, and also *LACS2* was observed, the differences in expression did not correspond consistently to adhesion quality as higher expression or lower (or no) expression occurred in cultivars with both good and poor husk adhesion. Expression of the Arabidopsis *WIN1/SHN1* gene, to which *NUD* is homologous, is thought to upregulate cuticular lipid biosynthesis in Arabidopsis (Aharoni et al., 2004; Broun et al., 2004; Kannangara et al., 2007; Oshima et al., 2013). In barley, virus-induced gene silencing of *HvWIN1* resulted in reduced expression of cuticle biosynthesis genes, including *LACS2*, and decreased proportions of C_16_ and C_18_ free fatty acids which are precursors to VLCFAs (Kumar et al., 2016). However, in covered barley, expression of a functional *NUD* gene results in lower expression of putative cuticle biosynthetic genes compared to naked barley which does not have a functional NUD protein (Duan et al., 2015). We found no differences in *NUD* expression between the two growth stages assessed, but as *NUD* is known to be expressed in the testa of caryopses (Taketa et al., 2008), it is possible that *NUD* is required to be expressed earlier than the growth stages assessed here to allow time for regulation to be effected distantly. Downregulation of genes involved in cuticle biosynthesis before the cementing material is produced is contrary to expected given that the cementing material is likely to be composed of cuticular material that can be seen to be produced during GS 77 by the irregular interface between the cuticle and outer epidermal wall (Gaines et al., 1985; Hoad et al., 2016; Brennan et al., 2017a). By GS 75 we observed higher expression of the *MYB96* transcription factor which upregulates expression of *Lipid transfer protein 3* (*LTP3*) (Guo et al., 2013), *ECH* which is required for cuticular lipid secretion (McFarlane et al., 2014) and *BDG* which is important for cuticle structure and polymerization of cutin (Kurdyukov et al., 2006a; Jakobson et al., 2016), suggesting together with the electron micrographs that synthesis of cuticular lipids has in fact increased rather than decreased. Duan et al. (2015) suggested that downregulation of cuticle biosynthetic genes may be responsible for production of a non-functional, permeable cuticle which causes adhesion. It is possible that permeability is achieved by upregulating synthesis of specific cuticular compounds, rather than downregulation of total cuticle biosynthesis. However, it may be that the transcriptional cascade upregulating cuticle biosynthesis occurs before GS 75, explaining why expression of many of these genes was higher at GS 73. Additionally, isolation of RNA from all caryopsis tissues rather than only the testa where *NUD* is expressed, and the pericarp where the cuticle is biosynthesized, may have diluted small changes in gene expression.

These data indicate that differences in husk adhesion quality among barley cultivars correlate with subtle differences in the composition of the solvent-extractible surface lipids of the caryopsis, and involve regulation of the proportion of sterol and triterpenoid compounds. The outer layer of the caryopsis surface lipids comprises a cementing material with specialized properties allowing adhesion, and these results support that compositional changes in the cementing material facilitates adhesion, potentially through altered cuticle permeability. There is clear variation in the quality of husk adhesion among genotypes, and significant genotype by environment interaction for those genotypes without extreme husk adhesion phenotypes. The data indicate that for those cultivars with a strong environmental component to adhesion quality, it is likely that altered ripening times or physical interaction between floret organs and environmental variables such as water ingress contribute, in addition to cementing material composition.

## Data Availability

The datasets generated for this study can be found in ArrayExpress, E-MTAB-7429.

## Author Contributions

SH and WT conceived the study. PH, LR, and MB designed the experiments. MB carried out plant growth and sampling, and wrote the manuscript. MB and SM did the microscopy. MB and TS did the lipid analysis. PH and JM did the microarray analysis. MB and CT did the data analysis. All authors have read the manuscript, given comments and approved the final version of the manuscript.

## Conflict of Interest Statement

The authors declare that the research was conducted in the absence of any commercial or financial relationships that could be construed as a potential conflict of interest.

## Footnotes

1 www.ebi.ac.uk/arrayexpress
